# Tertiary lymphoid structures and B lymphocytes: a promising therapeutic strategy to fight cancer

**DOI:** 10.3389/fimmu.2023.1231315

**Published:** 2023-08-09

**Authors:** Laura Esparcia-Pinedo, Nuria Romero-Laorden, Arantzazu Alfranca

**Affiliations:** ^1^ Immunology Department, Hospital Universitario de La Princesa and Instituto de Investigación Sanitaria Princesa, Madrid, Spain; ^2^ Department of Medicine, Universidad Autónoma de Madrid, Madrid, Spain; ^3^ Medical Oncology Department, Hospital Universitario de La Princesa and Instituto de Investigación Sanitaria Princesa, Madrid, Spain; ^4^ Cátedra Universidad Autónoma de Madrid (UAM)-Fundación Instituto Roche de Medicina Personalizada de Precisión, Madrid, Spain; ^5^ Centro de Investigación Biomédica en Red Cardiovascular, CIBERCV, Madrid, Spain

**Keywords:** tertiary lymphoid structures, B cells, adaptive anti-tumor response, TLS modulation, immunotherapy

## Abstract

Tertiary lymphoid structures (TLSs) are clusters of lymphoid cells with an organization that resembles that of secondary lymphoid organs. Both structures share common developmental characteristics, although TLSs usually appear in chronically inflamed non-lymphoid tissues, such as tumors. TLSs contain diverse types of immune cells, with varying degrees of spatial organization that represent different stages of maturation. These structures support both humoral and cellular immune responses, thus the correlation between the existence of TLS and clinical outcomes in cancer patients has been extensively studied. The finding that TLSs are associated with better prognosis in some types of cancer has led to the design of therapeutic strategies based on promoting the formation of these structures. Agents such as chemokines, cytokines, antibodies and cancer vaccines have been used in combination with traditional antitumor treatments to enhance TLS generation, with good results. The induction of TLS formation therefore represents a novel and promising avenue for the treatment of a number of tumor types.

## Introduction

Tumors form a complex structure composed by cancer cells and the tumor microenvironment. This microenvironment constitutes a heterogeneous and dynamic system that is the result of the interactions among different components such as immune and stromal cells, blood vessels and the extracellular matrix (1). Depending on the nature of the tumor, the immune component may be composed by cell populations with diverse functional activities, including: myeloid cells (neutrophils, macrophages, antigen-presenting cells (APC)), lymphocyte subsets such as Th-17 and Th-1 cells, follicular T helper lymphocytes (Tfh), regulatory T cells (Tregs), CD8+ cytotoxic cells, and B cells. The majority of myeloid cells (neutrophils, M2 macrophages, mast cells), Th-17 and Treg cells are known to promote tumor development, while cytotoxic, Tfh and Th-1 cells have anti-tumor activities (2). Accordingly, the composition of tumor immune microenvironment plays a key role in the response to therapy and patient outcome. Tertiary lymphoid structures (TLSs) are aggregates of organized immune cells (T and B lymphocytes, plasma cells, APC, macrophages) that appear in areas of chronic inflammation as tumors. Recent studies have reported the association of TLSs with both a better prognosis and the response to immune checkpoint-blocking therapies in a variety of cancers (3–7), suggesting that the presence of TLSs is involved in the progression and immune control of tumor growth. However, the functions of B lymphocytes in tumor development still remain unclear. Some studies suggest that these cells could have a protective function, while other authors support the opposite hypothesis (8, 9).

Here, we summarize the mechanisms of TLS generation and function, and provide insight into the role of antitumor therapies in the development and modulation of TLS characteristics and their consequences in cancer biology.

## Characteristics and composition of tertiary lymphoid structures

TLSs, also known as tertiary lymphoid organs (TLOs) or ectopic lymphoid structures (ELSs) (10), are aggregates of immune cells located in non-lymphoid tissues. TLSs are not present in physiological conditions, and normally they arise as a consequence of chronical inflammation in processes such as: autoimmune diseases (11, 12), allograft rejection (13) and in tumors as ovarian cancer, non–small cell lung cancer (NSCLC), colorectal cancer (CRC), breast, stomach, and liver cancers, and melanoma. The number, composition and location of TLSs vary among different tumors (6, 7, 14–25).

Under certain conditions, TLSs are given specific names for example iBALT (inducible bronchus-associated lymphoid tissue), that are used to design TLS-like structures present in the lung that appear in allergies, chronic infections or autoimmune conditions (26, 27). Similar to other TLSs, opposite functions have been described for the iBALT: a protective role for iBALT was reported in some animal models of infection (28–30), whereas a deleterious activity was also reported for these structures in both human and animal models of chronical inflammation (26, 27) and graft rejection (26).

**Figure 1:**
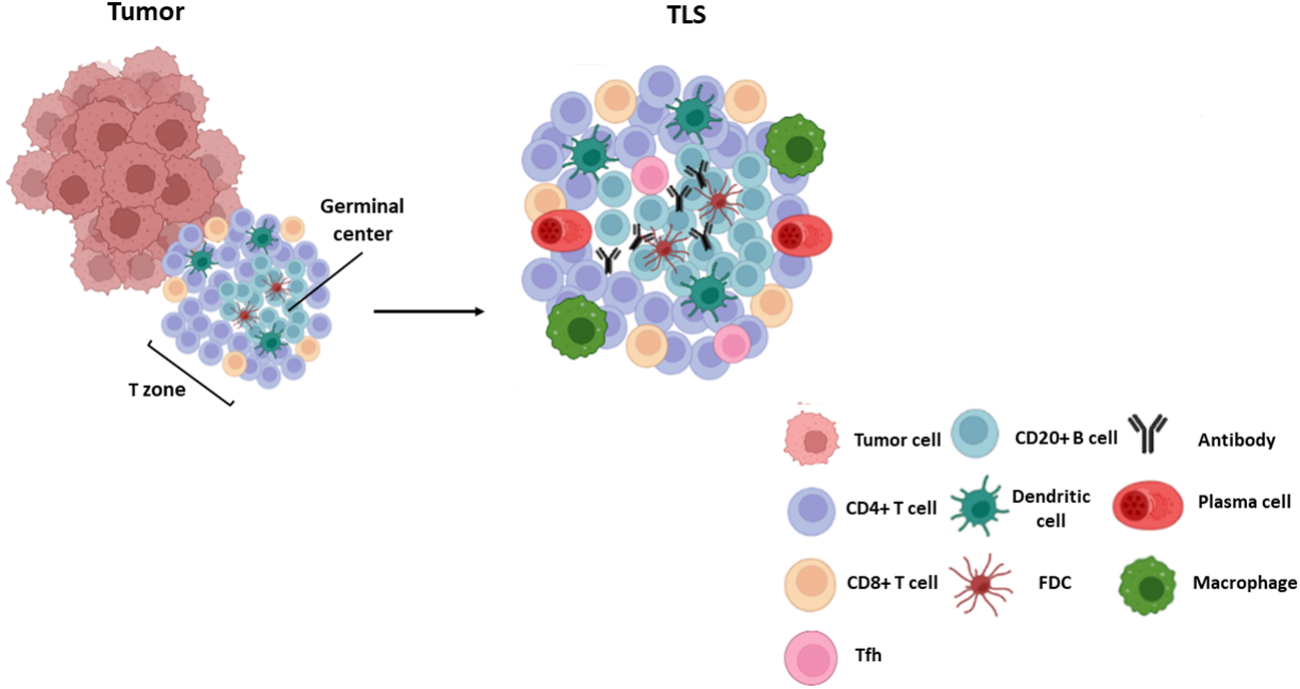
TLS composition. Mature TLSs are composed by a germinal center with B lymphocytes (CD20+ AID+ Ki67+ Bcl6+) and follicular dendritic cells (CD21+ CD23+) surrounded by a T zone composed by T follicular helper cells (Bcl6+ PD-1+ ICOS+ IL-21+), cytotoxic CD8+ T cells and CD4 + T lymphocytes. Other cells such as plasma cells (CD38+ CD138+), mature dendritic cells (DC-LAMP+) and macrophages (CD68+) are also found within these structures.

Cell composition and distribution in TLSs may differ among cancer types (1, 31), ranging from disorganized cellular aggregates -often referred to as immature or early TLS-, to well-organized and structured organs similar to SLOs. Mature TLSs typically comprise an internal B-cell zone, surrounded by a peripheral T cell-rich area, mainly composed by CD8+ cytotoxic T lymphocytes, CD4+Th-1 lymphocytes, follicular T helper lymphocytes, and LAMP3+ dendritic cells (31, 32) (**Figure 1**). Whereas mature primary TLSs are devoid of germinal reaction areas, secondary TLSs show clear active germinal centers with distinct dark and light zones within the B-cell area. The dark zone contains Ki67+ proliferating B cells that express BCL6 and AID (activation-induced deaminase), the enzyme that controls somatic hypermutation and Ig class switch. In the light zone, B lymphocytes are in contact with CD21+ and CD23+ follicular dendritic and T follicular helper (Tfh) cells that participate in clonal selection of B lymphocytes (**Figure 1**). These structural and functional characteristics point to TLSs as active sites of production of high affinity antibodies (32, 33). Recent studies have reported other subtypes of immune cells located in the T area, as plasma cells and macrophages (9, 34) (**Figure 1**). Recently, some authors have reported new markers for a better understanding of TLS composition. Some examples of the differences in TLS composition and selected markers are shown in **Table 1**.

**Table 1:** Examples of the TLS composition in different types of cancer in humans.

Cancer type	TLS Composition	References
**Non-small cell lung**	CD4+ and CD8+ T lymphocytes, Follicular dendritic cells (CD21+), mature dendritic cells (LAMP+) and plasma cells (CD138+)	(15–17)
**Breast**	CD4+ and CD8+ T cells, CD20+ B lymphocytes, Follicular dendritic cells (CD21+) and germinal centers (Bcl6+)	(35, 36)
**Gastric**	CD3+ T cells, CD20+ B lymphocytes (CD20+), plasma cells (CD138+), and follicular dendritic cells (CD21+).	(23)
**Ovarian**	CD20+ B cells and mature dendritic cells (LAMP+).	(37)
**Sarcoma**	CD8+ and Foxp3+ TReg lymphocytes, macrophages, CD31+ endothelial cells and CD20+ B cells.	(38)
**Skin**	Activated T lymphocytes, CD1a+ cells and mature dendritic cells (LAMP+).	(33, 39)
**Oral squamous cell** **carcinoma**	CD3+ T lymphocytes, CD20+ B cel, mature dendritic cells (LAMP+).	(40)
**Hepatic**	CD3+ T cells, CD20+ B cells, CD21+ dendritic cells.	(41)
**Pancreatic**	CD8+ T cells, CD20+ B cells, monocytes, Tregs	(42)
**Cell renal**	CD3+ T cells, CD20+ B lymphocytes, plasma cells (CD138+), and follicular dendritic cells (CD21+).	(5, 43)
**Melanoma**	CD3+ T cells, CD20+ B lymphocytes, germinal center (BCL-6+ and Ki67+) and follicular dendritic cells (CD21+) and mature dendritic cells (LAMP+).	(33)
**Colon**	CD3+ T lymphocytes, CD79+ CD20+ B lymphocytes, follicular dendritic cells (CD21+).	(18, 19)

In addition to mature TLSs, immune cell aggregates without a defined structure may be observed scattered within the tumor. Although these aggregates have been considered as immature forms of TLSs, some authors have proposed that they should be considered as different entities designated lympho-myeloid aggregates (LMA) (44). LMAs have been attributed specific anti-tumoral functions related with their cellular composition. Thus, plasma cell-rich LMAs may represent places of B-cell extrafollicular response, although their functions have not yet been fully elucidated. Likewise, disseminated clusters of CD20+ B cells in the proximity of T lymphocytes and macrophages are found in ovarian cancer, melanoma and other tumor types, which are thought to display either pro- or anti-tumoral activities. Finally, LMAs composed of T lymphocytes, macrophages or NKs have been hypothesized to act as active sites for anti-tumor responses. The relevance of LMAs, their relationship with tumor evolution and patient outcome, and the modulation of their composition and functional characteristics by antineoplastic therapies are issues of great interest that require further research.

## Formation of TLSs

The mechanisms of TLS development have not been fully elucidated. It is thought that initial events are similar to those of SLO formation, initiated by lymphoid tissue inducer cells (LTi), which are innate lymphoid cells characterized by the expression of the RORγt and ID2 transcription factors (45, 46). These cells secrete factors such as lymphotoxin α1β2 (LTα1β2) (38, 39) TNFα and IL-17 (47), which interact with their receptors on the surface of stromal cells known as LTo (lymphoid tissue organizers). Stromal cells in turn express adhesion molecules (ICAM1, VCAM1 and MADCAM1), and chemokines (CXCL12, CXCL13, CCL19 and CCL21), which regulate the recruitment of immune cells that participate in TLS formation from adjacent high endothelial venules (HEV) at later stages. These venules contain specialized endothelial cells that selectively express PNAd (peripheral lymph node addressin), a receptor for L-selectin, whose interaction is necessary for leukocyte trafficking to the TLSs (48). This assumption is supported by animal models where the deletion of IL-17Rα, CXCL13 or LTs impairs the development of TLSs. Likewise, the lack of RORγt, involved in LTi induction, also affects TLS generation (16, 49, 50).

**Figure 2:**
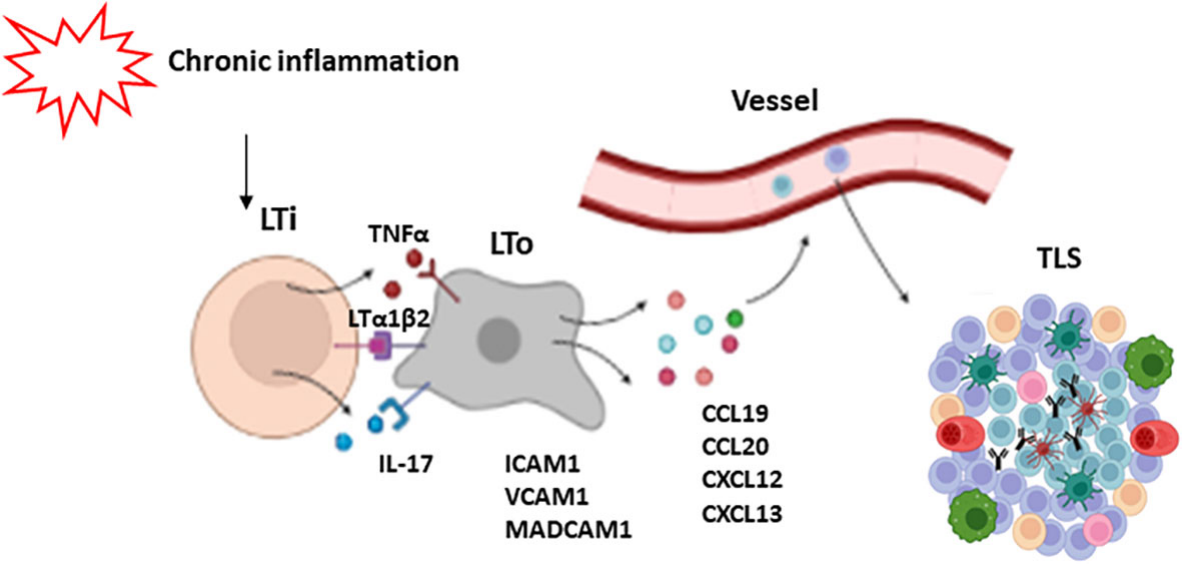
Mechanisms of TLS formation. Chronic inflammation leads to the activation of lymphoid tissue inducer cells (LTi). These cells secrete some factors such as LTα1β2, TNFα and IL-17 which activate LTo cells (lymphoid tissue organizers). As a response, LTo express some adhesion molecules like ICAM1, VCAM1 and MADCAM1, and chemokines like CXCL12, CXCL13, CCL19 and CCL21, which regulate the recruitment of immune cells that participate in TLS development.

Nevertheless, some authors have challenged the notion that LTis are actually necessary for the induction of TLS. Instead, other types of immune cells such as CD8+ T lymphocytes, Th17 cells, and M1-macrophagues among others, have been proposed as promoters of TLS development (51, 52). In addition, some articles highlight the relevance of fibroblasts, which are able to produce factors CXCL13, CCL19 and IL-17, as TLS initiators (53, 54). Similarly, other stromal and immune cells have been proposed as alternative LTo cells. Particularly, LT and TNFα signaling can promote the secretion by fibroblasts of certain chemokines such as CXCL13, CCL19 and CCL21, as well as B cell survival factors including BAFF, IL-7, and April (55). General mechanisms of TLS formation are depicted in **Figure 2**.

## TLSs in different types of cancers

Depending on the nature of the tumor, TLSs can be found in different proportions ranging from 10-20% in ovarian, hepatic and oral cancers (24, 40, 56) to 50% in breast cancer (35), NSCLC (17), melanoma (6) and gastrointestinal tumors (24), and more than 80% in colorectal cancer (21) and lung squamous cell carcinoma (57).

The presence of TLSs in tumors has been associated with variable outcomes. These structures may display either pro- or anti-tumoral activities, which are related to factors such as the number, composition and location of the TLSs. Higher densities of TLSs are related to a better prognosis in tumors including: ovarian (58), NSCLC (15), breast, renal cell, pancreatic (42), and hepatocellular (24) cancers, gastrointestinal tumors (59) and melanoma (6, 17). In this regard, in a murine model of metastatic melanoma, the use of a LTα fusion protein induces the formation of lymphoid structures in the tumor bed, where tumor antigen-specific lymphocytes are generated (60). Likewise, the proportion of mature dendritic cells in TLSs is highly associated with cytotoxic T cell responses and long survival in patients with non-small cell lung and rectal cancers (61, 62).

In contrast, in a murine model of hepatocarcinoma, incomplete TLS formation at the early stages of tumor development results in an impaired immune response and tumor growth. During tumor progression, TLSs may act as a niche for malignant progenitor cells, thus promoting tumor development (41). In addition, TLSs composed by high proportions of Tregs, which inhibit anti-tumoral responses, can promote neoplasia development. Indeed, tumor infiltration by Tregs has been linked to poorer outcomes in several types of cancer, including ovarian, breast and hepatocellular cancer (58, 63, 64). In support of these findings, another study demonstrated the presence of high proportions of Tregs in TLSs using a mouse lung adenocarcinoma model. Importantly, depletion of Tregs results in T cell proliferation and the triggering of anti-tumor responses (65).

**Figure 3:**
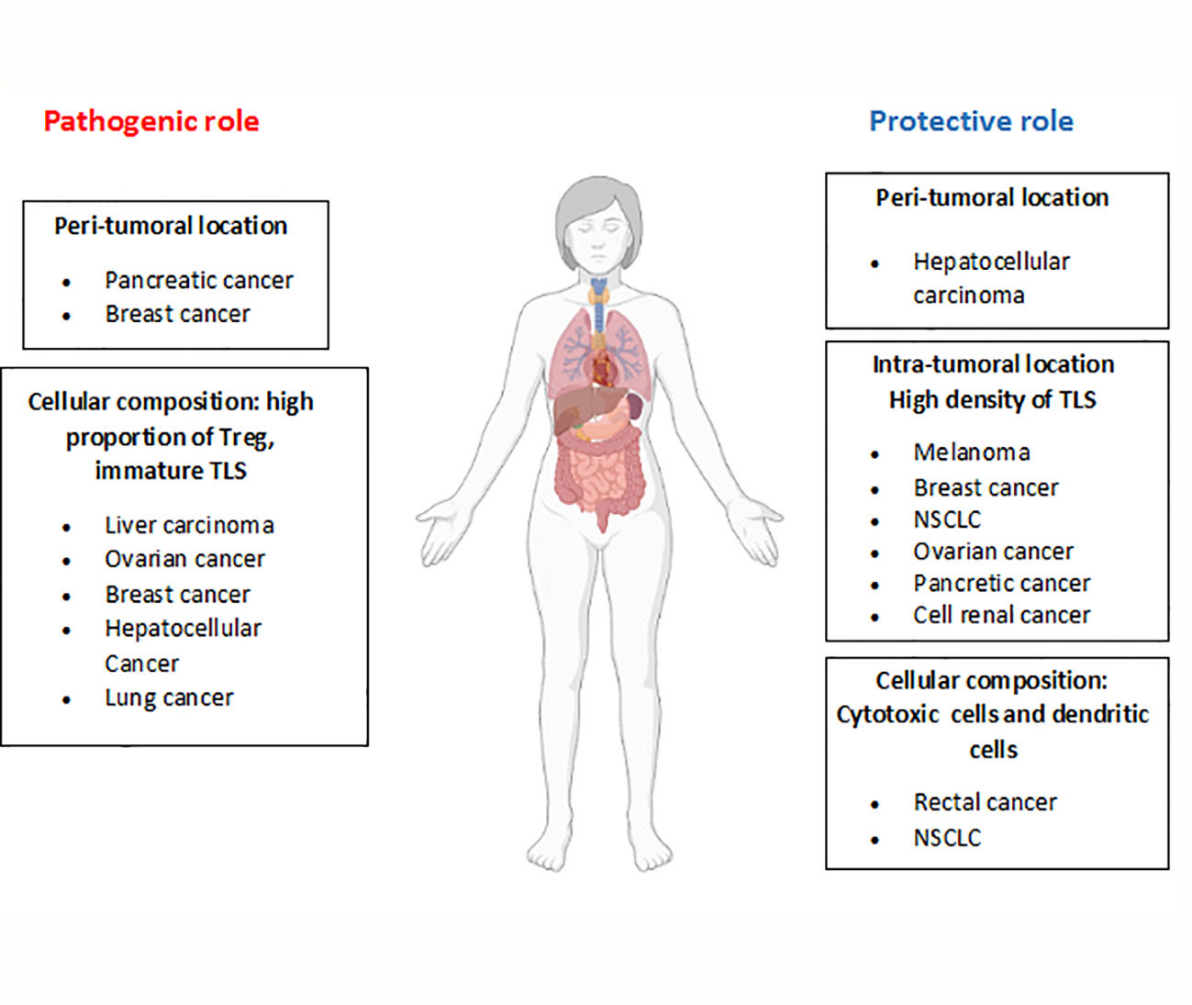
Dual role of TLSs in tumor development. Depending on the characteristics of TLSs (location, cellular composition, etc), opposite roles have been described for these structures in the development of different tumor types. The figure summarizes TLS features related to their pathogenic or protective role (bold letters) and the tumor types involved.

Depending on the location of TLSs (intra- or peritumoral), they may also exhibit different immune responses. While intratumoral TLSs have been associated with antitumor responses, peritumoral TLSs have been mainly related to protumor responses and poorer outcomes in patients with breast cancer, intrahepatic cholangiocarcinoma and pancreatic cancer (58, 65). Furthermore, in colorectal cancers, the presence of peritumoral TLSs is associated with advanced stages of the disease, tumor progression and worse prognosis (66–68). On the contrary, in hepatocellular carcinoma, peritumoral TLSs have been related to better outcomes (69). The mechanisms involved in this differential functionality remain unknown, but some studies in animal models have suggested that peritumoral TLSs may act as a pathway for cancer dissemination, resulting in worse prognosis (41), although this needs to be studied in depth. These findings are summarized in **Figure 3**.

## B cells in tumor TLSs

Different subsets of B cells can be found in the tumor microenvironment, mainly associated with TLSs. Mature B cells, plasmablasts and plasma cells have been found in several types of cancer, such as ovarian and prostate cancers (70), NSCLC (71) and renal cell cancer (43). Likewise, IgM+ plasma cells were detected in breast cancer and head and neck squamous carcinoma (72). Regulatory B cells (Breg), a special subset of B cells that play immunosuppressive roles, have also been detected in various types of cancer, such as prostate, gastric, hepatocellular and breast cancer (73–75). On the other hand, naive B cells were described to be very rare in some tumor types such as breast cancer (76), NSCLC (77) and melanoma (78).

The role of B cells in tumor biology has not been studied in depth, but again both pro- and antitumor activities were described for these cells (79, 80). B cells, however, can contribute to cancer eliminatation via tumor-associated antigen presentation or antigen transmission to dendritic cells, together with antibody production and ADCC (antibody-dependent cell-mediated cytotoxicity)/ADCP (antibody-dependent cell-mediated phagocytosis) phenomena. But the potential formation of immune complexes may increase inflammation, angiogenesis, and immunosuppression within tumor microenvironment, resulting in a poor outcome (81).

Most findings related to this question are based on mouse models. Thus, the protumor function of B cells was evidenced in μMT B cell-deficient mice, where reduced tumor growth was observed in MC38 colon carcinoma, B16 melanoma and thymoma (82). In the same line of evidence, another author reported a similar result after IgM antibody depletion in a murine melanoma model and in Rituximab-treated colorectal cancer patients (83). Opposite actions of B cells in tumor development may be partially explained by the existence of a heterogeneous population of varying composition, including subsets with immunosuppressive phenotype. Thus, recent investigations have described a Breg population composed of IL-10+ B cells that coexist with regulatory T cells (Tregs) in human breast and ovarian cancers, which favor the development of metastasis and promote tumor growth (84, 85). Interestingly, TGFβ production by Breg promotes both the polarization of CD4+ T lymphocytes toward Treg and the development of M2 macrophages cells in a mouse model of metastatic breast cancer, resulting in protumor activity. The observed effect in tumor development has been explained by the production of IL-10 by Tregs (86, 87). In addition, M2 macrophages synthesize C1q, a complement component that binds to the Fc portion of anti-tumor antibodies and triggers the classical component cascade in the presence of complement components such as C1r, C1s, C4, C2, C3 and C5 produced by tumor cells. These events result in the generation of some anaphylatoxins (C3a and C5a), which promote inflammation and angiogenesis. Similarly, B cells favor tumor growth by producing VEGF, which induces neovessel formation (88).

In the central areas of TLSs, however, B cells experience antibody class switching and somatic hypermutation, becoming plasma cells that produce tumor-specific antibodies (9) and may exert antitumor actions. In this regard, B lymphocytes in TLSs have been implicated in antitumor responses in patients with melanoma and ovarian cancer (37, 89). Similarly, in hepatocellular cancer, the proportion of B cells in TLSs directly correlate with the number of apoptotic tumor cells, and inversely correlate with the density of proliferating tumor cells (90).

## Function of antibodies in tumors

The ability of plasma cells from TLSs to produce specific antibodies (IgG and IgA) against tumor antigens has been demonstrated in multiple cancer types, such as NSCLC, breast cancer, and melanoma (36, 71, 91, 92). These cells may egress the TLSs and migrate into the tumor bed. In cancers with a higher proportion of TLS and plasma cells, specific antibodies can be detected surrounding the tumor, suggesting that such antibodies contribute to antitumor responses (43).

The identification of the antigens specifically recognized by intratumoral B lymphocytes is complex, and only a small number of antigens, mostly intracellular, have been described so far. These antigens are mainly related to proteins overexpressed by the tumor, or to tumor-specific posttranslational modifications or protein mutations. These antibodies may exert their anti-tumor functions by either direct inhibition, CDC (complement-dependent cytotoxicity), ADCC or ADCP. It is noteworthy that the frequent presence of autoantibodies in cancer patients, which seem to either pre-exist or arise by somatic hypermutation phenomena apparently favored under tumor conditions (44, 93). The isotype of autoreactive antibodies has been related to the prognosis of some types of cancer. Thus, in patients with breast cancer, ex vivo IgG autoantibody responses were associated with lower recurrence-free survival and reduced intratumoral CD8+ T-cell infiltrate, whereas IgA responses correlated with germinal center activity and increased number of TLSs (94). However, further research is needed to clarify the relative role of the different antibody isotypes in specific tumor settings.

Similarly, both isotype and tumor type may have an impact on anti-tumor activity of tumor antigen-specific antibodies. Thus, IgA can promote tumor development in mouse models of prostate and liver cancer whereas in human ovarian cancers, this isotype has a protective role. Accordingly, B cells produce IgA that binds to receptors universally expressed by ovarian cancer cells, sensitizing tumor cells to be eliminated by T cells and thus preventing tumor progression (92, 95). In contrast, immunosuppressive B cells in liver cancer are plasma cells expressing IgA, IL-10 and PD-L1. This phenotype depends on TGFβ-receptor (TGFβR) signaling (91). It is plausible that the presence of higher proportions of TReg expressing TGF-β whitin TLSs promote the generation of suppressive B lymphocytes, supporting the notion that TLS composition is determinant for anti-tumor activity.

Regarding IgG, it was described that elevated levels of IgG4 antibodies were associated with a poor prognosis in patients with melanoma (96), whereas in patients with NSCLC they were associated with a better outcome (97). IgG1 potentiates antitumor responses through the induction of T lymphocyte activity. Conversely, this isotype can exert a protumor function through complement activation (98), in particular at the C1q level. As a consequence, the production of C5a and C3a anaphylatoxins increases, fostering inflammation. Furthermore, these factors may promote angiogenesis and have been related to T cell depletion, thus favoring tumor progression (98, 99). In line with these findings, another report shows that *in vitro* formation of immunocomplexes may contribute to the suppression of cytotoxic activity (100). Hence, it is possible that the presence of immunocomplexes in the TME counteracts anti-tumor immune responses, resulting in the promotion of tumor growth.

## Modulation of TLS formation

**Figure 4:**
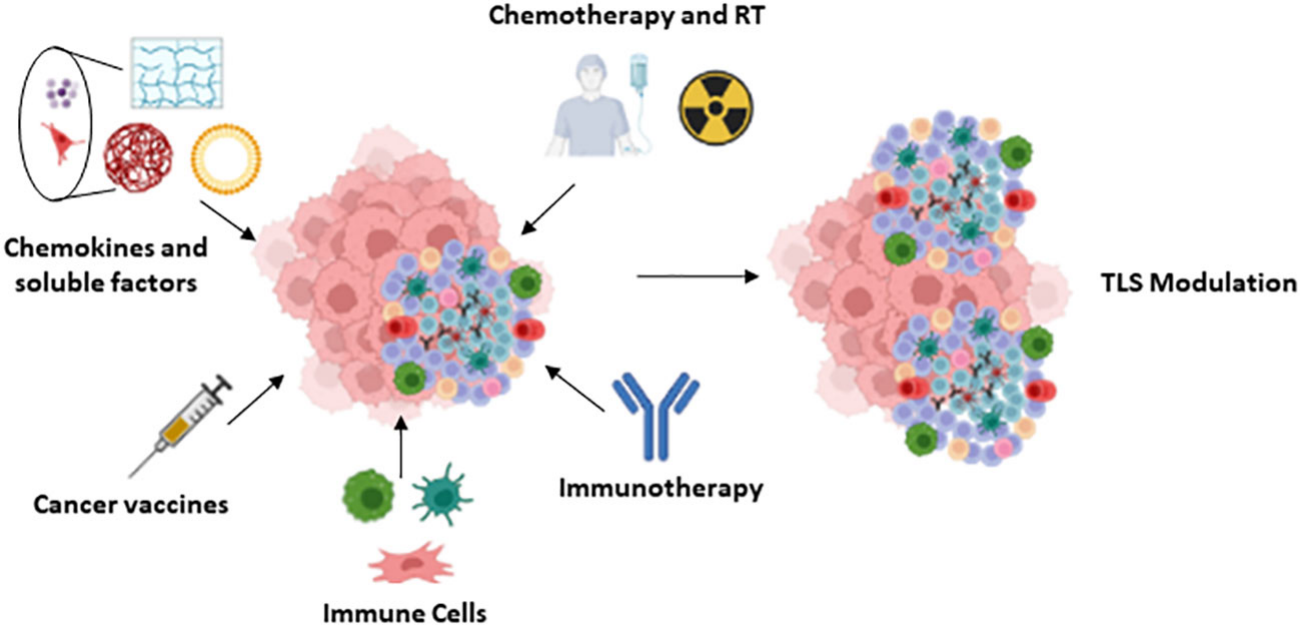
TLS modulation: Summary of the different approaches proposed for the modulation of the TLS.

As mentioned in previous sections, the number, location and composition of TLS, including the type of antibodies produced, may condition tumor progression and are associated with different patient outcomes. The modulation of these characteristics by specific strategies may therefore contribute to optimize antitumor therapy. Several approaches are currently underway to promote the formation of TLSs with anti-tumor action (**Figure 4**), some of which are detailed below.

## Chemotherapy

The impact of radiotherapy and chemotherapy agents on TLS development, as well as the potential role of these treatments on patient clinical outcome were recently investigated with contrasting results. For instance, radiotherapy locally induced the expression of MHC I and co-stimulatory molecules in a mouse model of breast cancer and increased CD8 + T cell infiltration into human tumors, which promoted an anti-tumor response (100). On the contrary, this treatment led to a decrease in the number of TLSs, followed by TLS restoration associated with a Treg enrichment in the TME (101). Similarly, in squamous cell lung cancer neoadjuvant chemotherapy impaired maturation of TLSs and germinal centers (57). Furthermore, in patients with lung squamous cell carcinoma and urothelial cancer, corticosteroid treatment used to control chemotherapy side effects reduced the number of TLSs, consequently leading to worse outcomes (33, 57, 102).

Conversely, some reports describe the induction of TLSs after neoadjuvant chemotherapy or radiotherapy in NSCLC, hepatoblastoma and pancreatic ductal adenocarcinoma, conferring a favorable prognosis (103). Interestingly, after radiotherapy in patients with breast cancer, tumors with higher densities of TLSs experienced an increase in tumor cell apoptosis (104). Other studies have shown that CXCL13 and Tfh and Th1 gene signatures were associated with improved responses in breast cancer patients treated with chemotherapy (105). In this line of evidence, another report based on a immunocompetent mouse model shows that neoadjuvant chemotherapy induces an ICOS-L+ B cell population that expresses the complement receptor CR2, which is associated with the development of TLS and improved disease-free and overall survival (106).

Cyclooxygenase-2 (COX-2) is a key mediator of pro-tumor responses, and is involved in the suppression of anti-tumor immunity, angiogenesis and proliferation of cancer cells (107–109). A study in prostate cancer revealed that COX-2 levels were reduced in the TLSs of patients with spontaneous cancer regression (110). Thus, therapies based on COX-2 blockade could be an attractive strategy to promote TLS-driven tumor immunity.

Mechanisms involved in the modulation of TLS formation by chemotherapy or radiotherapy have not been fully elucidated yet. TLSs experience cell apoptosis and transitory size reduction during radiotherapy. After several days, TLSs tend to normalize; however, the number of apoptotic cells still remains high, and CD8+/TReg ratio decreases during later stages, thus reflecting the immunomodulatory effect of radiation (101). Likewise, chemotherapy-induced tumor apoptosis promote an increase of phosphatidyl serine expression in the TME, which leads to a tolerogenic switch in immune infiltrate and subsequent tumor escape (111). Chemotherapy, however, may induce immunogenic cell death (ICD), that leads to the release of damage-associated molecular patterns (DAMPs) and potentiates immune antitumor response (112).

## Immunotherapy

Recently, different forms of immunotherapy have been used successfully to treat cancer. Among other effects, many of these treatments can modulate the formation and composition of TLSs. Thus, in a mouse model of breast and pancreatic tumors, the combination of anti-PDL1 therapies with antiangiogenic treatments can promote the development of TLSs and consequently the reduction of the tumor size. This was explained by the induction of HEV formation followed by TLS development and CD8+ lymphocyte activation, which resulted in tumor elimination (113). In line with these findings, the induction of TLSs was observed after the administration of anti-PD-1 in patients with lung cancer (114). Furthermore, cancer patients who responded to PD-1 and/or CTLA4 blockade had a higher basal number of TLSs with increased B-cell densities than non-responders (5, 7, 115, 116). In line with this, a recent multicohort phase 2 trial of pembrolizumab in combination with low-dose cyclophosphamide in patients with advanced soft-tissue sarcomas indicates that the presence of TLS is related to a better response to this therapy, especially in those patients in whom TLSs are not enriched in TReg (117). Another report demonstrates that an early response to anti-PD-1 antibody pembrolizumab in patients with different tumor types is associated with a gene signature related to Tfh cells (118), which are the main producers of IL-21 in the TME (119, 120). The blockade of this cytokine decreases B cell activation induced by the coadministration of anti-PD-1 and anti-CTLA-4 therapy (121). In addition, Th-1-oriented Tfh cells in TLSs are associated with higher expression of IL-21, and promote immunoglobulin and cytokine production after interaction with B and CD8^+^ lymphocytes, respectively (103). In line with these findings, a recent article in a mouse model of squamous lung cancer describes how anti-PD-1 increases circulating Tfh (cTfh), the number of TLSs and activation of B cells within these structures. Furthermore, anti-PD-1 induces CCL21 production in tumors, which may mediate cTfh migration to TLSs and subsequent B cell activation (122).

Cancer vaccines can also regulate the formation of TLSs. For example, an intradermal vaccine with an irradiated allogeneic granulocyte colony stimulating factor (GM-CSF), in combination with cyclophosphamide, promoted TLS formation in a cohort of 59 patients with pancreatic ductal adenocarcinoma (123). Furthermore, in patients with cervical neoplasia (CIN2/3), increased TLS development and maturation could be observed in regressing lesions after human papillomavirus vaccination (124). In these patients, vaccination increased the infiltration of CD8+ T cells, which secreted mediators that promoted the development of organized structures with germinal centers and T areas similar to TLSs, associated with HEV. Conversely, in unvaccinated patients the immune cells had a diffuse pattern distribution and were not organized into TLSs.

## Chemokines and soluble factors

The development of TLSs in tumors can be fostered by using molecules involved in the formation of these structures. For instance, LTβ receptor (LTβR) ligands promote the development of intra-tumoral TLSs associated with a protective role, and enhance survival in combination with immune checkpoint inhibitors in mice (125). Another report demonstrated that the expression of LTα in a murine model of pancreatic cancer increases CXCL13 and CCL21, resulting in the development of B and T zones within the TLSs. In this model, the expression of both molecules depends on the TNFR1 receptor, thus suggesting that signals through this receptor are sufficient for TLS formation (126). In addition, transgenic expression of IL-6 and its receptor gives rise to the increase of CXCL13 levels, which promote TLS development (127). Furthermore, TLR1, TLR2 and TLR4 ligands were also used for TLS neogenesis in several mice models of cancers (128–130). These molecules belong to the toll like receptor family, and are expressed in the surface of both innate immune cells and some non-immune cells like fibroblasts and epithelial cells. Signals triggered by the activation of these receptors lead to the activation of immune responses through the secretion of cytokines and chemokines. Thus, TLR ligands may represent a promising strategy to promote TLS-based anti tumor immune responses.

LIGHT (tumor necrosis factor superfamily member 14, TNFSF14) is expressed by activated immune cells (131), and binds to lymphotoxin beta receptor (132), which is present in stromal, epithelial and myeloid cells (133). This cytokine is known to play a key role in anti-tumor responses (134). Thus, the combination of LIGHT with an anti-VEGF antibody promotes T cell migration to the tumor, reducing tumor resistance to checkpoint blockade treatments (135). In addition, LIGHT induces the production of pro-inflammatory cytokines and chemokines such as IL-1β, IL-6 and CCL21, which recruit immune cells and enhance TLS development. In this regard, a fusion protein of LIGHT with a vascular targeting moiety (LIGHT-VTP) induces TLS formation in a mouse model of pancreatic cancer (125). Likewise, the combination of LIGHT-VTP with anti-VEGF and immune checkpoint inhibitors increase the amount of T cells and the number of HEVs in glioblastoma and lung metastases (136, 137).

Different approaches have been proposed to selectively deliver soluble factors to the tumor and minimize side effects secondary to systemic administration. One of these strategies is based on the use of collagen matrices containing therapeutic molecules. Successful results were obtained in mice that developed functional TLS in response to artificial collagen matrix with a thymus-derived stromal cell line that expressed LTα (138). The functionality of these TLSs was also proven by transplantating these structures in SCID mice, which developed a secondary immune response (139). Likewise, a collagen sponge scaffold containing gel beads with LTα1β2, CCL19, CCL21, CXCL12, CXCL13, and soluble RANK ligand, was implanted in mouse kidneys, and the presence of TLSs with defined T and B cell areas, follicular dendritic cells, and HEV could be demonstrated (140). An additional option is to use hydrogels, that have the ability to take up water and retain it (141). *In vitro* models have shown that hydrogel containing stromal cells that express BAFF and IL-4 increase the amount of B cells and promote class-switch events (142). Nanoparticles also represent a useful strategy to induce TLS formation. One of the advantages of this approach is that the release of the molecules contained inside nanoparticles can be tightly controlled, either through chemical properties of nanoparticle components or by cargo liberation under specific environmental conditions: e.g. pH, oxygen tension, protease activity, and so on. In addition, nanoparticles may be directed to specific structures or cell types by the addition of selected targeting molecules. Different types of nanoparticles were developed, such as liposomes, micelles or nanoparticles made from polyesters. These nanoparticles have the capacity to deliver different factors such as cytokines, chemokines, antigens, RNAs, etc (143–145).

## Immune cells

Some studies support the application of modified immune cells to induce the generation of TLSs within tumors. For instance, in a mouse model of sarcoma, the use of dendritic cells expressing T-bet promotes lymphocyte infiltration, Th1 responses and the development of TLSs, resulting in a reduction of tumor growth. This effect is mediated by the secretion of IL-36γ from Tbet-expressing dendritic cells (146, 147). This cytokine induces the expression of adhesion molecules and chemokines such as IL-8, CCL2 and CCL20 in endothelial cells, promoting T cell migration (148). A recent study in human colon cancer has shown that tumor IL-36γ associates with increased amount of central memory CD4+ T cells and a higher TLS number (149). In line with these results, DCs expressing chemokines involved in secondary lymphoid tissue development rapidly lead to increased T cell infiltration and enhance intratumor immune responses when injected into melanoma tumors (150). In addition, dendritic cells promote LTβR-dependent HEV differentiation and lymphocyte recruitment in the lymph nodes (151), and constitute a source of chemokines (CXCL12, CCL20/21,CCL19) involved in TLS development in the lungs of influenza virus-infected mice (152). In human lung cancer, autologous dendritic cells expressing CCL21 reduce tumor size, and increase the expression of some cytokines and chemokines like IFNγ, IL-12, and CXCL10 among others (153, 154). Several clinical trials are currently ongoing to evaluate the safety of a vaccine based on CCL21-expressing dendritic cells in different cancer types (155, 156).

Other cell types may be utilized to promote TLS formation. Human mesenchymal stem cells stimulated with TNF-α and IL-1β express higher levels of CCL19, VCAM-1 and ICAM-1, and induce CD4+T cell proliferation. These cells may act as LTo, generating the inflammatory environment required for TLS formation (157). Furthermore, intratumoral inoculation of stimulated LIGHT+ macrophages is sufficient to promote the development of TLS in a mouse model (125). These macrophages express high levels of CCL21 and TNFα, both of which enhance TLS formation in mice. Finally, in a model of infection with Salmonella, resident macrophages expressing CXCL13 and CX3CR1 promote the infiltration of B and CD4+ T cells in areas of infection, where they are activated, giving rise to TLS induction (158). Although these studies prove the involvement of these cell types in TLS generation, further studies are required to establish their suitability in a clinical setting.

## Concluding remarks

TLSs are structures where effective adaptive immune responses against tumors take place. In many cases, their presence is associated with improved response to therapy. Furthermore, most antineoplastic agents modulate TLS development, suggesting a role for these structures in the antitumor activity of these treatments. As such, TLSs have arisen as potential targets for the design of novel therapeutic approaches against cancer. However, TLSs may also be associated with poorer tumor progression and patient prognosis, so that the use of these structures as a therapeutic tool may have unintended deleterious effects. The factors involved in the differential role of TLSs are not fully elucidated, and include tumor type, TLS location within the tumor, specific cellular composition, and so on. The characterization of the specific mediators that regulate these characteristics is, therefore, a prerequisite for the optimization of treatments aimed at enhancing TLS-dependent anti-tumor immune response in an efficient and safe manner. Likewise, a careful selection of the target tumor type is necessary for the successful implementation of this strategy. The use of combinations of soluble factors that promote both TLS development and suitable cell composition, or the employment of selected immune cells modified to produce specific cytokines or chemokines, may contribute to the formation of TLSs with effective anti-tumor activity. In addition, the optimization of transporter systems that enable the delivery of mediators selectively to the tumor after systemic administration may facilitate their use in these patients, especially in barely accessible tumors and metastatic disease.

## Author contributions

LE-P, NR-L, and AA had scientific discussion for this work and wrote the manuscript. All authors contributed to the article and approved the submitted version.
